# Low-Intensity Pulsed Ultrasound for Delayed Union of Distal Radius Fracture After Palmar Locking Plate Fixation: A Case Report

**DOI:** 10.7759/cureus.51468

**Published:** 2024-01-01

**Authors:** Shunpei Hama, Koji Moriya, Yoshiyuki Matsuyama, Yutaka Maki, Hiroaki Nakamura

**Affiliations:** 1 Department of Orthopedic Surgery, Niigata Hand Surgery Foundation, Seiro-machi, JPN; 2 Department of Orthopedic Surgery, Tokyo Metropolitan Bokutoh Hospital, Sumida-ku, JPN; 3 Department of Orthopedic Surgery, Osaka Metropolitan University Graduate School of Medicine, Osaka, JPN

**Keywords:** ulnar variance, low intensity pulsed ultrasound, palmar locking plate, delayed union, distal radius fracture

## Abstract

Delayed union and non-union of distal radial fractures (DRFs) are rare, and there are a few reports of delayed union and nonunion of DRFs after palmar locking plate (PLP) fixation. A 68-year-old female patient presented to our hospital with left-sided wrist pain. Radiographs and computed tomography revealed a displaced DRF and ulnar styloid fracture. We performed open reduction and internal fixation with a PLP for the DRF and tension band wiring for the ulnar styloid fracture. However, bone union was not completed three months after the operation. We initiated low-intensity pulsed ultrasound (LIPUS) to achieve fracture healing. Complete bone union was confirmed radiographically five months after LIPUS. There have been few case reports on the delayed union or nonunion of DRFs after PLP fixation treated with LIPUS. LIPUS might be an effective option for the delayed union of DRFs after PLP fixation.

## Introduction

Nonunion of distal radial fractures (DRF) is extremely rare [1], and there are a few reports of delayed union and nonunion of DRFs after palmar locking plate (PLP) fixation [2,3]. These cases are treated with revision surgery using an autogenous graft or demineralized bone matrix using PLP fixation. There are few case reports of delayed union or nonunion of DRFs after PLP fixation treated by low-intensity pulsed ultrasound (LIPUS). However, a multicenter, prospective, randomized double-blind, placebo-controlled study reported accelerated healing of fresh DRF enrolled in the study within seven days after the fracture [4]. Herein, we report a case of delayed union of the DRF after PLP fixation, which was treated with LIPUS. The patient provided informed consent for the publication of this case report and any accompanying images, and the study was approved by our institutional review board.

## Case presentation

**Figure 1:**
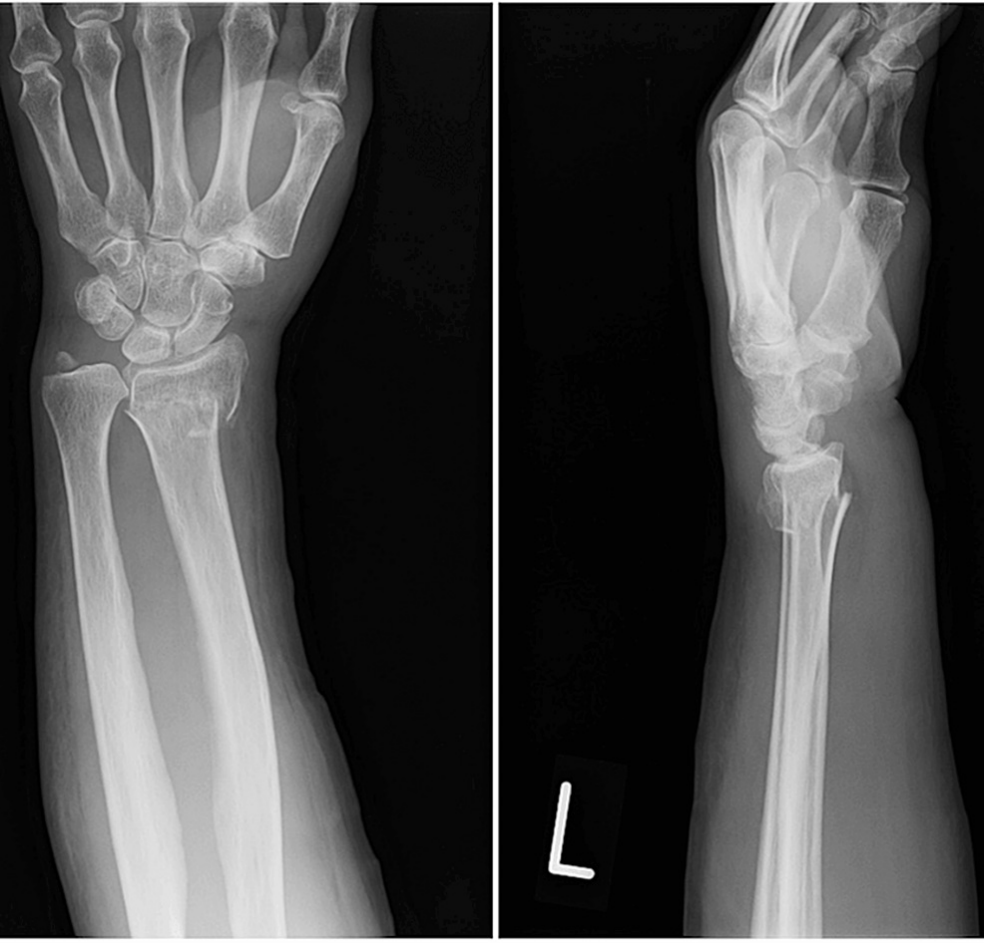
Preoperative posteroanterior and lateral radiographs of the left wrist.

**Figure 2:**
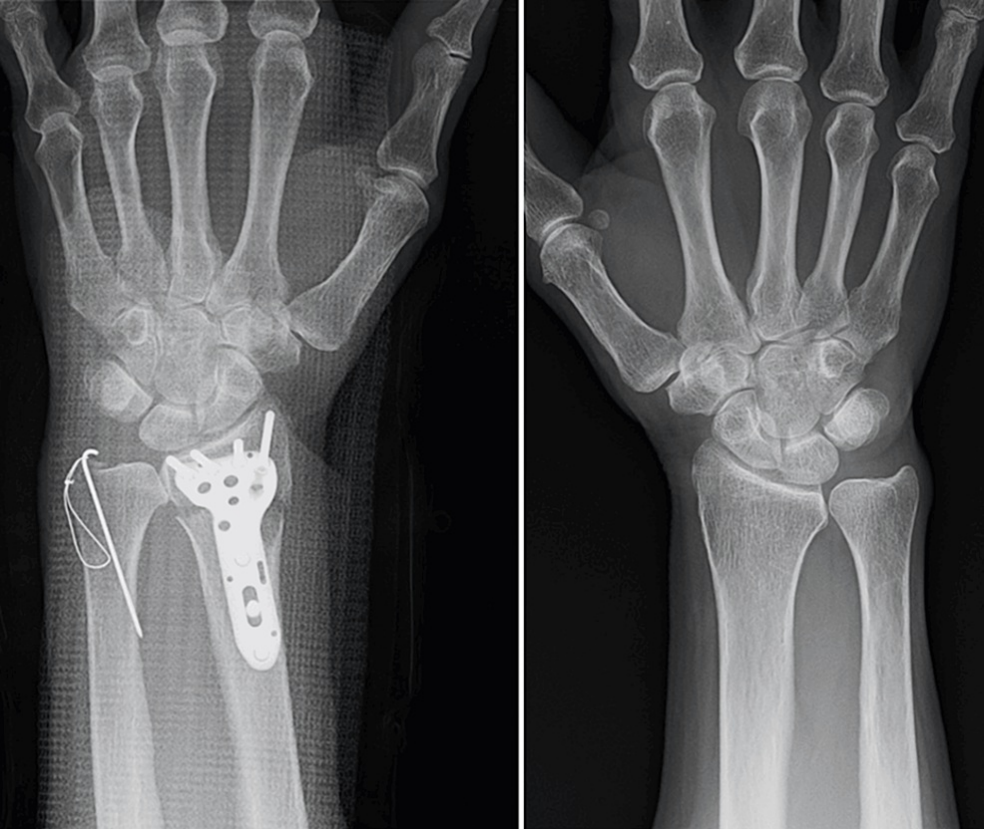
Posteroanterior radiographs of the affected side after the surgery and the intact side.

A 68-year-old left-handed woman was referred to our hospital because of a left wrist injury. Physical examination revealed swelling and tenderness in the left wrist. Radiographs and computed tomography (CT) images of the left wrist revealed a displaced DRF and ulnar styloid fractures (Figure 1). Five days after the injury, we performed open reduction and internal fixation (ORIF) using the PLP system and tension band wiring for DRF and the ulnar styloid fracture, respectively. Radiographs of the right unaffected wrist showed 2.7 mm ulnar plus variance, while the ulnar variance of the affected wrist after the operation was 0 mm ulnar plus variance (Figure 2).

**Figure 3:**
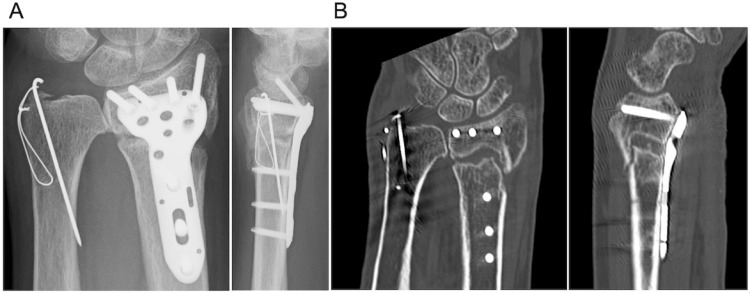
(A) Posteroanterior and lateral radiographs of the left wrist three months after the surgery. (B) Coronal image and sagittal computed tomography images three months after the surgery.

**Figure 4:**
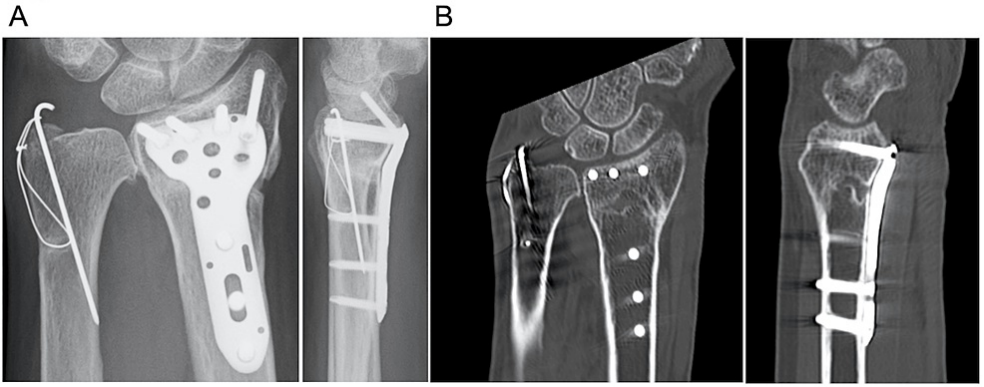
(A) Posteroanterior and lateral radiographs of the left wrist 5 months after the application of low-intensity pulsed ultrasound (LIPUS). (B) Coronal and sagittal computed tomography images 5 months after the application of LIPUS.

Three months after surgery, radiographs and CT showed delayed union of the DRF (Figures 3A, 3B). The patient was also diagnosed with osteoporosis. The bone mineral density T-score of the femoral neck was -2.3. In her blood test, procollagen type 1 N-terminal propeptide was 90.5 μg/L, and tartrate-resistant acid phosphatase isoform 5b levels were 485 mU/dL. The patient refused reoperation for delayed union and preferred less-invasive treatment. LIPUS was initiated for 20 min a day from the radial side of the fracture site. Bone union was completed five months after LIPUS (Figures 4A, 4B). Eight months after the surgery, she was completely pain-free, and the grip strength of her left hand was 20 kg. The range of motion of the left wrist was equal to that of the contralateral side. The Disabilities of the Arm, Shoulder, and Hand score was 11.4 points.

## Discussion

In the present case, the delayed union was treated with LIPUS. There have been few case reports on the delayed union or nonunion of DRFs after PLP fixation treated with LIPUS. In a review of 46 articles by Margaliot et al., which included 917 patients treated with external fixation (EF) and 603 patients after ORIF for unstable DRF, the rates of nonunion after EF and ORIF were reported to be 0.1% and 0.0%, respectively [5]. There are two cases reported on the nonunion and delayed union of DRFs after PLP fixation [2,3], in which one was treated with a demineralized bone matrix graft and internal fixation using the same type of PLP [2], whereas the other case was treated with autogenous iliac crest bone graft and internal fixation using different types of PLP [3]. We believe that immediate therapeutic intervention is necessary for the delayed union because one patient experienced redisplacement and plate breakage at the original fracture site 14 weeks after PLP fixation for DRF [2].

 This patient had no underlying conditions that could be construed as the cause of the delayed union, such as diabetes, morbid obesity, peripheral vascular disease, alcoholism, or smoking habits. Excessive distraction of the fracture has been reported as a cause of nonunion after EF [6] and ORIF using PLP [3]. In the present case, the ulnar variance of the affected wrist after the operation was 0-mm ulnar plus variance, although there was a 2.7-mm ulnar plus variance in the unaffected wrist. In addition, an osteoporosis examination was performed, since a fragility fracture was suspected. Her BMD T score was -2.3SD and her TRACP-5b was elevated. She was diagnosed with osteoporosis after the operation, but the osteoporosis did not influence the progress of significant fracture healing in the distal radius [7]. We aimed for a 0-mm ulnar plus variance because 0-mm or 1-2-mm ulnar positive variance is a reasonable goal for reduction in middle-aged and elderly patients with osteoporosis [8]. However, neutral ulnar variance caused excessive distraction of the fracture for the patient.

 A controversial point of this case report is that the union of the fracture site might have been completed without LIPUS. However, healing of the delayed union appeared to be accelerated radiographically after applying LIPUS. Kristinasen et al. reported the efficacy of LIPUS for fresh DRFs which had been treated with manipulation and a cast within seven days after the injury [5]. In the study, the time to union of the group treated by LIPUS was significantly 38% shorter than that of the placebo group. In the study, LIPUS was applied from a cast window created in the dorsal aspect. In the present case, the delivery of LIPUS from the radial side of the fracture site, because the callus formation of the radial side was poorer than that of the dorsal side.

## Conclusions

In conclusion, we believe that LIPUS might be an effective and suitable option for the delayed union of DRFs after PLP fixation. We recommend LIPUS for patients who refuse reoperations or those who are not suitable for it. In addition, assessment of the unaffected side of radiographs is important to avoid the excessive distraction of the fracture site when performing PLP fixation for DRF.
